# Sixth European Seminar in Virology on Virus–Host Interaction at Single Cell and Organism Level

**DOI:** 10.3390/v10080400

**Published:** 2018-07-28

**Authors:** Elisa Saccon, Adriana Vitiello, Marta Trevisan, Cristiano Salata, Giorgio Palù

**Affiliations:** Department of Molecular Medicine, University of Padova, 35121 Padova PD, Italy; elisa.saccon.1@phd.unipd.it (E.S.); adriana.vitiello89@gmail.com (A.V.); marta.trevisan@unipd.it (M.T.); cristiano.salata@unipd.it (C.S.)

**Keywords:** organoids, transcriptomics, virus, in vitro models, in vivo models

## Abstract

The 6th European Seminar in Virology (EuSeV) was held in Bertinoro, Italy, 22–24 June 2018, and brought together international scientists and young researchers working in the field of Virology. Sessions of the meeting included: virus–host-interactions at organism and cell level; virus evolution and dynamics; regulation; immunity/immune response; and disease and therapy. This report summarizes lectures by the invited speakers and highlights advances in the field.

## 1. Introduction

The 6th European Seminars in Virology (EuSeV), organized by the European Society for Virology (ESV), was held at the University of Bologna Residential Center in Bertinoro (Bologna, Italy), on 22–24 June 2018. The topic of this edition of the EuSeV was “virus–host interaction at single cell and organism level”, innovative approaches to understand how viruses utilize host cell functions for their multiplication, and how viruses are held in check by immunity at the cellular and systems levels. The 6th EuSeV was organized in five sessions: virus–host-interactions at organism and cell level; virus evolution and dynamics; regulation; immunity/immune response; and disease and therapy. The invited speakers discussed different virus–host systems, ranging from latent and persistent to lytic infections. Participants explored how host factors that restrict or facilitate specific steps of infection can be identified, and how RNA viruses dynamically use pro- and antiviral factors in the course of error-prone replication. Deep knowledge of virus–host interactions provides the basis for the development of antiviral therapies as well as the opportunity to understand cell functions, based on the notion that viruses represents excellent biological probes to study cell biology.

The meeting hosted advanced graduate students, post-docs, and established scientists from all the world, and provided a free and interactive atmosphere for scientific exchange and discussion. The program of the meeting and abstracts of invited and selected participants are available at the web site http://www.eusev2018.de.cool/. This article summarizes lectures by the invited speakers and highlights advances in the field presented at the conference.

## 2. Meeting Report

The presentations of the invited speakers of the 6th EuSeV have been summarized in the following sections according to the scientific program.

### 2.1. Virus–Host-Interactions at Organism and Cell Level

#### Chairs: Gabriella Campadelli-Fiume, Giorgio Palù, Dana Wolf, and Ben Berkhout

- Marcela Pasetti and Nicholas Zachos (Dept. of Pediatrics, Center of Vaccine Development, University of Maryland, College Park, MD, USA; Johns Hopkins University, Baltimore, MD, USA): Modeling rotavirus infection and maternal immunity in human enteroids.

Stem cell-derived organotypic models or organoids are in vitro 3D models that have been developed for multiple organs (e.g., intestine, lung, brain, etc.) and closely recapitulate the in vivo situation in terms of heterogeneity and organization of cell types. Indeed, organoids represent a new exciting experimental model to study virus-host interactions and pathogenesis. In this context, Marcella Pasetti reported the development of a co-culture model based on adult or pediatric enteroid monolayers—derived from human intestinal biopsies, and characterized cellular morphology, physiological, and biochemical features—with the addition of macrophages and neutrophils to generate the immune enteroid environment. Interestingly, this model of enteroid monolayers are permissive to rotavirus infection and exhibit cytotoxicity features similar to those seen in humans [1]. Furthermore, using pediatric enteroids, she reported data about the benefits of human breast milk on the intestinal mucosa. In fact, human breast milk breast milk inoculated in enteroids lumen leads to an increase of transepithelial resistance and occludin in tight junctions. Breast milk also enhances defensin 5 and has an impact on other immune components such as granulocyte-macrophage colony–stimulating factor. Overall, this lead to a protection from viral infection.

- Katja Wolthers (Dept. of Medical Microbiology AMC Amsterdam, Amsterdam-Zuidoost, Amsterdam, The Netherlands): The age of organoids: new ways for virus host interaction studies.

Organoids are in vitro models that could revolutionize virus research that currently relies heavily on 2D cell culture and animal models, which have limited potential for studying human–pathogen interaction. Katjia Wolthers highlighted this concept, showing the contrast in clinical and animal studies along with genomic diversity and strain adaptation to laboratory (and animal) culture that has been reported in the attempt to understand the enterovirus A71 (EV-A71) pathogenesis in humans. EV-A71 is an important human pathogen and it has been associated with hand, foot, and mouth disease, as well as a variety of neurological diseases including aseptic meningitis, encephalitis, and poliomyelitis-like paralysis. Katjia Wolthers described the development of organoids of gut and lung that represent excellent models of the entry sites of EV-A71 in humans. Analysis by real time (RT)-qPCR and virus titration assay reveal strain specific replication kinetics that correlate with data from clinical isolate already characterized [2]. By studying viral capsid proteins, they were also able to observe that VP1-145 is controlling the switch to P-selectin glycoprotein ligand-1 (PSGL-1) receptor binding. Overall, these results highlight the potential for 3D cell culture models to increase our understanding of virus pathogenesis, providing a better alternative for the use of 2D culture cell or animal models.

- Angela Ciuffi (Institute of Microbiology, Lausanne University Hospital and University of Lausanne, Lausanne, Switzerland): Single-cell analyses applied to HIV.

Advances in the combination antiretroviral therapy (ART) against HIV infection have greatly contributed to improve patient survival and quality of life. However, ART does not eradicate the virus, because of the early establishment of a long-lived viral reservoir, which represents one of the main barriers to a successful cure. Although many cell types can be infected by HIV and may constitute part of the latent reservoir, CD4+ T cells represent the best-characterized reservoirs for HIV in virally suppressed subjects on long term ART. Different CD4+ T cell subsets can be infected with HIV and be present at multiple cellular states, either resting or activated [3]. With the purpose of targeting viral reservoirs, one approach is the so-called “shock and kill” strategy, which aims at reactivating HIV particle production from latent cells so that they will be killed by virus-mediated cytotoxicity or by cytotoxic CD8+ T lymphocytes, thereby leading to the eradication the latent reservoirs [4]. Angela Ciuffi described how reactivating latent infected cells, by means, for example, of stimuli leading to T cell receptor (TCR)-mediated cellular activation, can lead to a heterogeneous population of cells, where some are successfully induced while some others remain unresponsive. Indeed, to better understand transcriptional programs leading to successful reactivation of HIV expression, Ciuffi’s group employed an established HIV latency model that uses human primary CD4+ T cells and exposes them to different reactivation conditions (such as the previously mentioned TCR or hydroxamic acids vorinostat (SAHA), a histon deacetylase inhibitor). Then, they analyzed the transcriptomic profile using single-cell RNA sequencing, to characterize transcriptional heterogeneity during HIV latency and reactivation. The results showed that latently infected cells are transcriptionally heterogeneous and can be separated in two distinct cell clusters, according to distinct transcriptional profile. Moreover, the different HIV reactivation phenotypes, induced and non-induced, overlap with transcriptionally defined cell clusters, where one cluster is represented by poorly responsive cells and thus is not successfully induced, while the other cluster presents high levels of HIV transcripts and corresponds to induced cells. Indeed, these findings confirm that latently infected cells display different degrees of resting depths that correlate with their level of global HIV transcription; cells from cluster 1 are in a deeper resting state, and are more difficult to activate upon TCR stimulation; while cells from cluster 2 are in a less deep resting state and are more responsive to cellular activation and to HIV expression reactivation. Overall, their analysis identified transcriptional programs involved in a successful reactivation of HIV expression, which could provide a valuable tool to facilitate the identification of powerful latency reversing agents, able to stimulate HIV expression in all the resting cells regardless of their phenotypes, and to help identify potential biomarkers of inducible cells [5].

- Urs Greber (Institute of Molecular Life Sciences, University of Zurich, Zurich, Switzerland): Mechanisms in cell-to-cell variability of virus infection.

Virions are particles largely inert, but when they interact with host cells, they become dynamic, promoting the infectious process. The molecular events and the outcome of a virus infection are variable between cells. This is due to heterogeneity in receptor abundance, endosomal and cytoplasmic trafficking, virion uncoating, the transcription of viral genes, the production and stability of viral proteins, the assembly of progeny particles from preformed components, and the egress of virions from the infected cell. All these steps depend on the complexity of the molecular interactions between the virus and host. Urs Greber gave a lecture focalized on human adenovirus (HAdVs) infection to understand the complexity and functionality of lipidome, highlighting the high levels of ceramide sphingolipids yield in cells after viral penetration by endocytosis [6]. Then, starting from the lipid homeostasis, he reported data about the alteration of protein homeostasis, in particular the activation of the unfolded protein response (UPR) that induce endoplasmic reticulum (ER) stress promoting HAdV spreading [7].

- Ben Berkhout (Dept. of Med. Microbiology, Academic Med. Center of the University of Amsterdam, Amsterdam, The Netherlands): Humanized mouse model of HIV.

In the absence of an effective vaccine and lack of a complete cure, HIV infection remains a worldwide burden, with more than 35 million people infected (UNAIDS 2016). Therefore, there is an urgent need for improved, potentially curative HIV therapeutic regimens, and the research necessary to develop these interventions requires extensive preclinical evaluations in robust, reproducible, and reliable models of HIV infection. Cell culture systems and ex vivo tissue explants have been used extensively to model HIV infection, however, HIV infection can be best replicated using in vivo models, due to the huge complexity of the dynamics of HIV transmission and disease progression in humans, which cannot be simulated in vitro [8]. Ben Berkhout described some applications of humanized mice for better understanding HIV infection and pathogenesis, as well as for testing new antivirals. Indeed, HIV has a very limited species tropism that prevents the use of most conventional small animal models for AIDS research. Whereas one could consider the SIV- or SHIV-macaque model, this is expensive, raises ethical concerns, and non-human lentiviruses still retain some differences compared with HIV [9]. For these reasons, different humanized mouse models have already been set up and employed in different research fields on HIV. For example, BLT mice have been extensively used to study HIV persistence/latency, and killing strategies, in an effort to eradicate HIV-infected cells [10]. The group of Berkhout has established a human immune system (HIS) mice by transplanting newborn BALB/c Rag2-/-IL-2Rγc-/- immunodeficient mice with human hematopoietic stem cells transduced with a doxycycline-inducible lentiviral vector. They thus developed a new inducible gene therapy approach for the hematopoietic system, to be used, for instance, as a preclinical tool to test efficacy and safety issues [11]. HIS mice are a valuable tool also for testing new antiviral drugs, such as BDM2, a very potent antiretroviral agent, comparable to the best anti-HIV drugs currently on the market, with full activity against viruses resistant to all current drugs. BDM2 is currently been tested in a Phase I safety clinical trial. Finally, Berkhout explained the usefulness of this in vivo model for the evaluation of an anti-HIV gene therapy approach, which combines four short hairpin RNAs (shRNAs), targeting conserved regions of the viral genome, within a single lentiviral vector. Indeed, the preclinical safety profile of this new combinatorial platform was first evaluated in HIS mice in view of further development towards clinical trial phase I [12].

- Karin Metzner (Department of Infectious Diseases and Hospital Epidemiology, University Hospital Zurich, Zurich, Switzerland): Unravelling HIV-1 latency, one patient at a time.

Upon HIV-1 infection, the viral genome is permanently integrated into the host cells DNA, resulting in few infected cells, mainly CD4+ T cells, persisting as latent viral reservoirs. This small population of cells does not actively produce progeny virions and is not affected by either antiretroviral therapy or the host’s immune system. Karin Metzner explained how single-cell omics technologies (i.e., genomics, epigenomics, transcriptomics, epitranscriptomics, proteomics, and metabolomics), complemented with dedicated bioinformatics pipelines, can offer new opportunities to investigate HIV-1 pathogenesis, especially focusing on HIV-1 latency. Indeed, understanding how HIV-1 can persist despite the current antiretroviral treatment could represent a crucial point to develop strategies for the eradication of the viral reservoir. Moreover, single-cell omics profiles could be used to uniquely identify latently infected cells and predict possible outcomes of the patient’s treatment. To achieve this goal, Metzner is collaborating with other research groups in a Swiss interdisciplinary project, called HIV-X. The researchers involved in HIV-X are studying a group of approximately 1600 individuals who have been treated successfully with antiretroviral therapy for at least five years, and whose viremia has been suppressed to below the detection limit over this period of time. The scientists, then, are analyzing the decay rates of the viral reservoir, as well as host and viral genomes of these individuals, using state-of-the-art technologies, such as droplet digital PCR (ddPCR). Indeed, ddPCR was found to be a reliable method to quantify proviral and episomal HIV-1 DNA targets well below the detection limit of quantitative real time PCR, thus making ddPCR particularly well suited to measure the size of HIV-1 latent reservoir and suggesting that this assay could prove useful for clinical studies [13]. Finally, the HIV-X study’s complex datasets will be used to define host and viral factors that have an impact on the mechanisms of HIV-1 latency and develop models, which could be used to predict how a patient responds to a particular treatment. In doing so, the scientists working on this project also hope to contribute to the development of more effective treatments that might eliminate the viral reservoirs for good [14].

- Massimiliano Pagani (Department of Medical Biotechnology and Translational Medicine, Università degli Studi di Milano, Milano, Italy): Single cell approaches to study the immune system.

The single cell multi-omics combined with imaging technologies that track dynamics and spatial organization is now inspiring a new vision of understanding the progression of human diseases, at cellular resolution and genomic breadth. Using this new approach, Massimiliano Pagani described his work on the characterization of cancer development looking at the cancer immunesurveilance to understand the mechanism of escape that allows the progression of the disease. In particular, he focused on the presentation of the strategies to improve the therapeutic efficacy against the tumor infiltrating regulatory T lymphocytes (Treg) cells that are a potent suppressor of effector cells. Through Treg depletion, it is possible to enhance the anti-tumor specific immune responses and reduce the tumor burden. In particular, he reported data obtained using the single cell analysis to observe the differences in transcript gene expression in both normal and tumor tissues, thus identifying a new molecular signature as a therapeutic target that could be used to predict clinical responses to immune therapies [15].

- Brad Rosenberg (Icahn School of Medicine at Mount Sinai Annenberg Bldg. 17-70C, New York, NY, USA): Single cell transcriptomics for characterizing the human immune response to yellow fever virus.

Systems immunology methods have demonstrated great utility for studying immune responses to different vaccines and pathogens. Transcriptomics techniques serve as core components of systems-level characterizations and are often applied to complex mixtures of immune cells such as peripheral blood mononuclear cells (PBMC). Such “mixed cell” transcriptional profiles present challenges in associating gene expression (and corresponding function) with specific cell types, and can also obscure functionally significant contributions by rare cells. However, single-cell RNA sequencing has recently emerged as a powerful tool for mapping cellular heterogeneity in diseased and healthy tissues. In particular, the recently developed high throughput droplet microfluidics RNA-seq strategies have the potential to overcome the issues of the “mixed cell” transcriptomics approaches [16]. Brad Rosenberg described the inDrops RNA-seq methodology and its application to characterize the human immune response to yellow fever vaccine in PBMC at single cell resolution. His results have revealed cell type-specific gene expression programs, in both innate and adaptive components, in response to this highly effective and clinically important vaccine.

### 2.2. Virus Evolution and Dynamics

#### Chairs: Michael Kann and Esteban Domingo

- Esteban Domingo (Centro de Biología Molecular Severo Ochoa (CSIC-UAM), Campus de Cantoblanco Universidad Autónoma de Madrid, Madrid, Spain): Confronting RNA viruses: from quasispecies to lethal mutagenesis.

The talk of Esteban Domingo focused on RNA viruses and their elevated mutational rate during viral replication. This process gives rise to a complex spectrum of mutant species, called quasispecies. This highly complex and dynamic population structure may have profound implications in viral evolution and pathogenesis, and understanding its behavior could improve the control of viral disease, especially in terms of finding multi-epitopic vaccines and combination therapies [17]. Indeed, a new antiviral strategy, called “lethal mutagenesis”, is based on the idea that within-host viral populations can be driven to extinction when the load of deleterious mutations is artificially increased with a mutagen, and becomes too high for the population to be maintained. This concept derives from one of the equations of the quasispecies theory; there is an error threshold within population dynamics that discriminates the maintenance of genetic information. Indeed, this correlates with the notion of extinction threshold for RNA viruses; when mutations have an adverse average impact on fitness, the mean fitness of a population sets to a dynamic equilibrium between selection, which improves equilibrium and mutation, which degrades it. This dynamic equilibrium is called mutation–selection balance. If the equilibrium mean fitness is too low, the population size decreases deterministically to extinction [18]. Effective antivirals, for lethal mutagenesis therapy, require additional agents able to mutagenize the virus, but not the cell, and to provide an advantage over standard non-mutagenic inhibitors and their combinations. Among them, Favipiravir has proven to be a potent mutagenic agent against many RNA viruses, such as influenza virus, West Nile virus, Ebola virus, and hepatitis C virus (HCV) [19]. Moreover, in a recent work, Esteban Domingo evaluated the adaptation, mutant spectrum dynamics, and phenotypic diversification of HCV during 200 passages in human hepatoma cells, in an experimental design that precluded coevolution of the cells with the virus. By investigating genetic and phenotypic changes occurring upon prolonged passage of HCV, he reported that the virus exhibited internal population disequilibria, in terms of phenotypic traits, that did not decline with increased adaptation to the host cells. The diversification suggests that perturbations within the viral populations may provide a selective advantage to viruses that can be fully exploited in changing environments [20]. Overall, these studies can provide valuable tools for the development of new effective antivirals, able to control or prevent the development of viral resistance and escape.

- Marco Vignuzzi (Viral populations and Pathogenesis Unit, CNRS UMR 3569, Institut Pasteur, Paris, France): Monitoring, predicting, and targeting virus evolution and transmission.

RNA viruses introduce many mutations in their genome during replication, due to an error-prone copying of virus genomes by the viral RNA polymerase. Mutations often result in non-sense genetic information that can lead to a dramatic decrease of viral fitness, but can also sometimes lead to an enhancement of virus infection, transmission, or escape from the immune responses. Marco Vignuzzi described this mutated progeny as a “mutant swarm”, a cloud of variants, or quasispecies, that are similar but distinct from one another and have a different ability to survive to new environmental conditions, thus resulting in an evolution selection of one set of variants over the others. With the next generation sequencing (NGS) technologies, it is possible to identify all the mutants in a virus population, however, potent bioinformatic platforms are required for analyzing the huge amount of data generated. Thus, Marco Vignuzzi and his group developed a computational pipeline, ViVan (virus variance analysis), that facilitates the characterization of the mutant swarm and helps monitor the changes in mutant populations [21]. Moreover, by combining mathematical algorithm and predictions to NGS data, it is possible to identify biological signals to monitor, and possibly predict, RNA virus evolution [22]. Vignuzzi then introduced the concept of “sequence space”, which is a multidimensional framework that represents all of the possible variants in a population, and how each of those mutations relate to each other. However, among these variants, the biologically relevant are few. Thus, by applying mathematics to experimental data and developing ways to reduce the dimensionality of viral sequence space, each variant could be located in different point of a landscape, according to their fitness topology, represented as mountains (high fitness) and valleys (low fitness) to a 2D map. Vignuzzi’s expectation is that if we are able to reconstruct empirical fitness landscapes, we could monitor a population’s evolutionary movement and potentially predict the direction it will take.

### 2.3. Regulation

#### Chairs: Veronika von Messling, Lynn Enquist, Rebecca Dutch and Angela Ciuffi

- Lynn W. Enquist (Department of Molecular Biology, Princeton University, Princeton, NJ 08544-1014, USA): Silenced or productive infection? Engagement of an alphaherpesvirus with peripheral nervous system neurons.

Alpha herpesviruses infections establish a life-long infection in the nervous systems of infected humans and animals and can reactivate upon various stress signals. Remarkably, in the natural host, infection of epithelial cells with these viruses results in productive infection (litic infection), whereas infection of peripheral nervous system neurons results in non-productive silent infection (latent infection). Of note, infection of dissociated peripheral neurons in culture also results in productive infection. To study the molecular mechanisms of escape from latency, Enquist’s group used primary neurons cultured in compartmented tri-chambers in which the cellular soma and neurite compartments are divided. In this way, they recapitulated the natural route of infection by infecting axons with low dose of virus resulting in a latent infection in a small number of neuronal cell bodies. Using these cultures, they developed a new complementation assay to investigate the molecular signals, leading to escape from latency and establishment of productive infection. Enquist’s group found two different mechanisms of silencing escape: a cellular stress mediated-slow route, which requires protein kinase A and c-Jun N-terminal kinase activity, and a viral tegument protein mediated-fast route that is independent of the host cell kinases activities. Overall, the results showed that the induction of a productive infection in neurons, even at a low multiplicity of infection, requires either the presence of tegument proteins or the activation of the protein kinase A (PKA) and c-Jun N-terminal kinase (JNK) pathway [23].

- Rebecca Dutch (Dept. of Molecular & Cellular Biochemistry, University of Kentucky, Coll. of Med., BBSRB Bldg., Lexington, KY 40536-0509, USA): Entry, replication, and spread of negative strand viruses: lessons from human metapneumovirus.

Human metapneumovirus (HMPV), a paramyxovirus identified in 2001, is a leading cause of respiratory tract infection particularly in children, immunocompromised patients, and the elderly. HMPV infections account for an estimated 10% of respiratory tract infections and have incidence rates comparable to those of influenza virus infections during winter. Nonetheless, no specific antiviral drugs or vaccines protecting from HMPV infections are currently available, and clinical treatments are mainly supportive [24,25]. Rebecca Dutch presented her group’s work on viral entry and their findings on viral replication. She reported that many aspects of HMPV infection differ from those of previously characterized, closely related viruses. For instance, the HMPV fusion (F) protein is sufficient to promote viral attachment and entry, with the help of the viral attachment protein, and entry involves binding to the attachment factor heparan sulfate and internalization of the virus [26]. After attachment and endocytosis, viral nucleocapsids are released into the cell cytoplasm. Incoming particles can give rise to replicative sites that fuse into larger inclusion bodies, located close to cell nuclei. From inclusion bodies, viral RNA might be transported to assembly sites through the actin cytoskeleton, requiring a dynamic process that needs to be elucidated completely [27]. Finally, paramyxovirus spread generally involves assembly of individual viral particles, which are released to infect new target cells. In contrast, Rebecca Dutch showed that infection of human bronchial airway cells with HMPV results in formation of intercellular extensions and of extensive networks of branched cell-associated filaments, which depends on actin polymerization. Co-culture assays supported the hypothesis that viral spread from infected to new target cells occurs through these extensions, with viral proteins and RNA detected in these structures, suggesting direct transfer of viral genetic material to new target cells [28]. Overall, these findings have revealed novel mechanisms for entry, replication, and viral spread, opening new possibilities for antivirals and vaccine development.

### 2.4. Immunity/Immune Response

#### Chairs: Monsef Berkirane, Urs Greber, Marlène Dreux and Richard E. Randall

- Marlène Dreux (CIRI, Inserm U1111 CNRS UMR 5308, Lyon, France): Flavor of flavivirus by plasmacytoid dendritic cells.

The innate immunity is an essential defence mechanism of the host cell to viral infections. In particular, type I interferon (IFN-I) signaling is pivotal for the host control of viral infections, nonetheless, many viruses evolved mechanisms to evade this response in infected cells. Marlène Dreux introduced the new alternative sensing pathway of the innate immunity mediated by plasmacytoid dendritic cells (pDCs), which involves physical cell–cell contact with infected cells. pDCs are a rare group of immune cells that act as sentinels of RNA viral infection, recognizing evolutionary distant viruses. pDCs produce high levels of type I interferon and, when in contact with infected cells, they establish a strong response that induce their polarization and activation through the production of antiviral molecules. Cells polarization includes local accumulations of cell adhesion molecules, modified lipids, and proteins known to modulate membrane dynamics and trafficking, along with cortical components. The endocytosis machinery also polarizes at contact, in agreement with their previous report revealing the cell-to-cell transmission of immunostimulatory RNA to pDCs via exosome-like vesicles. Marlène Dreux proposes that the pDC/infected cell contact site represents a novel form of synapses specialized for vesicular-mediated transmission of viral immunostimulatory RNA, named “interferogenic synapse”. In particular, she demonstrated that in the presence of Dengue virus (DENV) or Chikungunya virus (CHIKV), the TLR7-induced signaling in pDCs promotes cell polarity and establishment of sustained contacts, highlighting positive feedback regulations [29]. A model system in which IFN-I response is pDC-restricted has been developed to probe pDC antiviral and immunomodulatory functions. The results demonstrated that sensing of DENV and CHIKV infected cells by pDCs results in IFN-I production in the absence of other inflammatory cytokine responses. This pDC response leads to early control of both viruses and is sufficient to protect mice from CHIKV-induced lethality. Interestingly, the early pDC activation also results in an accelerated type II IFN response, via the activation of natural killer (NK) cells. Collectively, IRF7-mediated pDC activation orchestrates IFN-I and II responses controlling arbovirus infections.

- Richard E. Randall (University of St. Andrews, School of Biology, BMS Bldg., North Haugh KY16 9ST, UK): Paramyxoviruses, interferon, and persistence; variations at the molecular, viral, cellular, and organism levels influence the outcomes of infection.

Paramyxoviridae is a large family of single stranded, negative sense RNA viruses that cause a variety of serious illnesses in humans and animals, ranging from respiratory infections to diseases such as measles, mumps, and encephalitis (e.g., Nipah virus). Richard Randall described his studies on how these viruses interact with the interferon (IFN) system; how they trigger an IFN response; how, once induced, interferon stimulated gene products inhibit virus replication; and how paramyxoviruses, at least partially, circumvent the IFN response. Randall reported examples showing that the way different paramyxoviruses, and different isolates of the same virus, interact with the IFN system, at the molecular, cellular, and organism levels, strongly influences the outcome of infections. It has been reported that some paramyxoviruses can establish persistent infections [30], and in his presentation, Randall proposed a model in which this occurs. He showed that, at late times post-infection, parainfluenza virus type 5 (PIV5) transcription and replication can be repressed in either an IFN-dependent or IFN-independent manner, leading to the establishment of persistent infection in tissue culture cells. In this condition, PIV5 fluxes between active and repressed states within individual cells. Interestingly, single amino acid substitutions in different PIV5 isolates determines whether or not virus replication can be repressed, suggesting that during early acute phases of virus infection PIV5 variants, in which virus replication is not switched off, might be selected as these viruses will have a selective replicative advantage. Nevertheless, as the infection progresses, viral variants in which replication can be repressed will be selected as they may establish prolonged/persistent infections also in the context of a developing adaptive immune response.

### 2.5. Disease and Therapy

#### Chairs: Monique Lafon and Thomas Mertens

- Monique Lafon (Department of Virology, Institut Pasteur-Paris, Paris, France): Takeover by rabies virus G protein of signaling pathways driving neuron survival: a source of innovative therapeutical molecules for neurodegenerative diseases.

Rabies is a neurological disease caused by a rhabdovirus of the genus Lyssavirus. The disease affects domestic and wild animals, and is spread to people through close contact with infectious material (such as saliva), via bites or scratches. After replicating in muscles, the rabies virus (RABV) gains access to the peripheral nervous system before entering the central nervous system (CNS) by a process of retrograde axonal transport. RABV spreads rapidly to the brain, resulting in an invariably fatal encephalitis. The success of viral transmission in new hosts depends on the preservation of the neuronal network, allowing viral replication and transmission through secretion of viral progeny into the salivary glands. To this end, RABV has developed two main mechanisms to escape the host defences based on the following: (i) its ability to kill protective migrating T cells and (ii) its ability to enter into the NS without triggering apoptosis of the infected neurons and preserving the integrity of neurites. The tolk of Monique Lafon focused on the discovery that during RABV infection, the viral envelope G protein (RABV-G) triggers neuron survival by affecting the specific phosphorylation of the phosphatase and tensin homolog (PTEN) protein mediated by the microtubule-associated serine and threonine kinase 2 (MAST2) protein [31]. In particular, Lafon showed that a peptide (referred to as “Neurovita”) constructed from RABV-G induces neuro-protective and neuro-regeneration in different types of neurons and in preclinical animal models. Indeed, Neurovita offers promising perspectives in the field of regenerative medicine of the nervous system.

## 3. Discussion and Conclusions

The 6th EuSeV has been an actively participated meeting with extensive discussions that involved senior and young scientists. During the scientific sessions, participants highlighted the importance of new technologies and model systems and, in particular, the high potentiality of the organoids as a model system to better understand viral pathogenesis. Although organoids could overcome the numerous limitations of the animal models, it is essential to demonstrate that they can fully recapitulate the natural process of viral infection. It is, in fact, foreseen that combining the new omics technologies at single cells level with the organoid models will allow one to achieve a better characterization of the molecular mechanisms that regulate the virus–host interaction and the development of viral diseases.
